# Classification and prediction of *Mycobacterium Avium* subsp. *Paratuberculosis* (MAP) shedding severity in cattle based on young stock heifer faecal microbiota composition using random forest algorithms

**DOI:** 10.1186/s42523-021-00143-y

**Published:** 2021-11-14

**Authors:** Alexander Umanets, Annemieke Dinkla, Stephanie Vastenhouw, Lars Ravesloot, Ad P. Koets

**Affiliations:** 1 Department of Bacteriology, Host Pathogen Interaction and Diagnostics Development, Wageningen Bioveterinary Research, Lelystad, The Netherlands; 2 Department of Population Health Sciences, Faculty of Veterinary Medicine, Utrecht University, Utrecht, The Netherlands; 3 Present Address: Chair Group Youth Food and Health, Faculty of Science and Engineering, Maastricht University Campus Venlo, Venlo, The Netherlands

**Keywords:** *Mycobacterium avium* subsp. *paratuberculosis*, Gut microbiota, Machine learning, Prediction, Pathogen shedding, Bovine, Random forest

## Abstract

**Background:**

Bovine paratuberculosis is a devastating infectious disease caused by *Mycobacterium avium* subsp. *paratuberculosis* (MAP). The development of the paratuberculosis in cattle can take up to a few years and vastly differs between individuals in severity of the clinical symptoms and shedding of the pathogen. Timely identification of high shedding animals is essential for paratuberculosis control and minimization of economic losses. Widely used methods for detection and quantification of MAP, such as culturing and PCR based techniques rely on direct presence of the pathogen in a sample and have little to no predictive value concerning the disease development. In the current study, we investigated the possibility of predicting MAP shedding severity in cattle based on the faecal microbiota composition. Twenty calves were experimentally infected with MAP and faecal samples were collected biweekly up to four years of age. All collected samples were subjected to culturing on selective media to obtain data about shedding severity. Faecal microbiota was profiled in a subset of samples (n = 264). Using faecal microbiota composition and shedding intensity data a random forest classifier was built for prediction of the shedding status of the individual animals.

**Results:**

The results indicate that machine learning approaches applied to microbial composition can be used to classify cows into groups by severity of MAP shedding. The classification accuracy correlates with the age of the animals and use of samples from older individuals resulted in a higher classification precision. The classification model based on samples from the first 12 months of life showed an AUC between 0.78 and 0.79 (95% CI), while the model based on samples from animals older than 24 months showed an AUC between 0.91 and 0.92 (95% CI). Prediction for samples from animals between 12 and 24 month of age showed intermediate accuracy [AUC between 0.86 and 0.87 (95% CI)]. In addition, the results indicate that a limited number of microbial taxa were important for classification and could be considered as biomarkers.

**Conclusions:**

The study provides evidence for the link between microbiota composition and severity of MAP infection and shedding, as well as lays ground for the development of predictive diagnostic tools based on the faecal microbiota composition.

**Supplementary Information:**

The online version contains supplementary material available at 10.1186/s42523-021-00143-y.

## Background

Bovine paratuberculosis (Johne's disease) is a widespread chronic wasting disease caused by *Mycobacterium avium* subsp. *paratuberculosis* (MAP) infection. The disease is extremely relevant in the context of dairy farm practice due to induction of significant economic losses related to the animal wasting, infertility and decrease in the milk production [1].

It is generally accepted that calves can be infected with MAP in the first days of life, however, the development of MAP shedding and clinical symptoms can take years and varies substantially between individuals in severity [2]. Faecal shedding of MAP into the environment has been shown to be high in animals with clinical symptoms [3]. Nevertheless, severe MAP shedding can occur even in asymptomatic animals, which in turn is correlated with an increased rate of transmission of paratuberculosis in a herd [4]. Therefore, timely identification of high shedding animals is essential for pathogen control and minimization of economic losses.

Widely used methods for detection and quantification of MAP, such as culturing and PCR based techniques, rely on the presence of MAP cells or its DNA in a sample and have little to no predictive value for disease development. In addition, despite a very high specificity of culturing and PCR based tests, their sensitivity can vary greatly due to the intermediate shedding of MAP cells by infected animals [5, 6]. In this light, the development of a diagnostic tool for populations with an endemic MAP infection that will allow prediction of MAP shedding severity without relying on the presence of the pathogen in a sample or a strong and specific immunological response is an attractive goal.

The pathophysiology of bovine paratuberculosis is complex as exposure of animals to MAP may lead to clearance of MAP (no infection) or infection. Infection of the distal part of the small intestine as the preferred site of infection leads to the formation of granulomatous lesions characterized by accumulation of macrophages in the lamina propria. These lesions can contain large amounts of MAP cells (multibacillary) of very few (paucibacillary). In some animals these lesions remain relatively stable for years while in others a progressive disease develops which will lead to intractable diarrhoea, proteins loosing enteropathy and ultimately death. The factors driving these changes and different outcomes are only partly understood and involve multiple factors including host immunity and genetics, MAP genetic factors as well as environmental factors (reviewed in Koets et al. [7]). In cattle, as a part of the infectious process, MAP colonizes the intestine and induces changes in the intestinal environment due to invasion into the mucosa [8], and is forced to interact with members of the intestinal microbiota. It has been shown in observational studies that MAP infection induces noticeable differences in the gut microbiota composition of affected cows [9, 10] In an experimental rabbit model alteration of microbiota by diet in turn also influenced the severity of MAP infection [11]. These findings indicate a bilateral relationship between MAP and other intestinal microorganisms.

The composition of the intestinal microbiota has been shown to be a biomarker for gastro-intestinal health and disease development. Microbiota composition analysis showed a potential for prediction of gastro-intestinal cancer [12], liver disease [13], and other not-communicative disorders [14–16]. However, the forecasting of a disease or disorder development using microbiota composition is not a trivial task. Microbiota composition in both humans and cattle have been shown to be relatively stable within a single individual [17, 18], however the composition shifts dramatically with the change of hosts diet [19] and age [20, 21] in cattle, lambs and humans. These changes introduce a high level of noise in the data that could render forecasting very difficult or inaccurate. Another challenge comes from the complex nature of microbiota composition data. Large datasets can be composed from hundreds of samples and each sample may contain a very divers microbial community. Complex, not normally distributed, zero inflated data can be challenging for statistical analysis and deciphering of the relevant signal. Nevertheless, machine learning approaches have shown a great promise in addressing challenges presented by the microbiota compositional data including prediction of host susceptibility to pathogens [22]. Machine learning is a branch of artificial intelligence that advanced considerably in recent years and is now commonly used in multiple aspects of everyday life as well as science. Machine learning is a broad term for a family of statistical techniques that are taking advantages of a large amount of data and computational power of modern computers to detect patterns in data [23]. Machine learning algorithms, and in particular Random Forest, became a popular solution for analysis of various types of microbiota data, due to its flexibility [24].

In the current study the feasibility of MAP shedding intensity prediction based on microbial composition differences between animals with different cumulative shedding of MAP cells throughout their life was investigated. The following study is a proof of concept study that aimed to lay the foundation for development of microbiota composition based diagnostic and prediction tools.

## Methods

### Animals

Details of the animal experiment have been previously described in detail in a PhD thesis [25] and in a publication by Ganusov et al. [26]. In short, twenty neonatal female Holstein–Friesian calves were purchased from several commercial high health farms in the Netherlands with a documented paratuberculosis unsuspected status and housed at the Wageningen Bioveterinary Research (WBVR) pathogen free facilities (Lelystad, The Netherlands). Calves were raised following best practices, on a diet with restricted access to a commercially available milk replacer and calf concentrate. In addition, calves had free access to hay and drinking water. Following weaning at 6 weeks of age the animals were fed with a conventional diet based on grass silage, concentrates and free access to water corresponding to their age and lactation status. The diet was never supplemented with fresh grass and animals were kept indoors for the duration of the experiment.

Practices of the animal husbandry during the experiment were closely resembling dairy cattle farming practices commonly accepted in the Netherlands except for outdoor grazing. Animals were bred around 15 months of age. In the second year of the study six animals were culled due to infertility and two due to development of pathological conditions not related to the experimental MAP infection. The remaining 12 animals survived for at least 50 months out of the total of 55 months of the experiment duration. During the experiment none of the animals developed any signs of clinical paratuberculosis (severe diarrhoea, weight loss, emaciation, oedema).

### Experimental infection and samples collection

A faecal suspension from a single donor-cow with clinical signs of paratuberculosis and consistently shedding MAP as confirmed by culture and MAP specific IS900 qPCR [27] was used as the inoculum. Every calf was orally dosed with 20 g of inoculum in 200 mL of commercially available milk replacer recommended for use in dairy calf rearing, three times per week for the period of four weeks. After the experimental infection of animals, faecal samples were collected every two weeks.

Samples were collected from the rectum using a clean set of disposable latex gloves per calf without lubricant. Samples were transported to lab immediately where a part was taken for direct MAP culture and quantification, the rest was rapidly stored for further analysis at − 20 °C as has been described previously [25].

### DNA extraction and amplicons library preparation

For microbiota profiling, a subset of samples was selected from the experimentally infected cattle. Two hundred sixty two samples were selected to represent the overall dataset with the particular focus on samples that were collected at crucial animal husbandry time-points: pre-weaning period (1 month), post-weaning period (3 and 7 months), start of sexual maturity (12 months) and start of first lactation (24 months); Additional file 1). Prior to DNA extraction faecal samples were thawed and 0.1 g of a sample was subjected to repetitive bead-beating (3 × 30 s with 5 s cooldown in between) using Lysing Matrix B (2 mL tube) and the FastPrep-24 instrument (MP Biomedicals). Total DNA was extracted individually from every faecal sample using QIAamp Fast DNA Stool Mini Kit (QIAGEN) according to the manufacture instructions. In short, following homogenization the samples were lysed in a lysis buffer, treated with InhibitEX to remove inhibitors from the sample, and proteins were digested using a proteinase K treatment. DNA was subsequently bound to a silica spin column, washed twice and eluted in a low-salt buffer. Extracted DNA was subjected to quantification using CLARIOstar Plus (BMG Labtech) and Quant-iT™ dsDNA Assay Kit, high sensitivity (ThermoFisher Scientific) according to instructions provided by the manufacturer.

Amplicon library preparation and sequencing were performed at BaseClear (Leiden, The Netherlands). The V3-V4 region of 16 s rRNA gene was amplified with 341F (5′-CCTACGGGNGGCWGCAG-3′) and 785R (5′-GACTACHVGGGTATCTAATCC-3′) [28] primers in a two-step protocol. Generated amplicons were appended with Illumina adaptors and sequenced on the Illumina MiSeq platform. The sequencing data was demultiplexed, Illumina adaptors were removed and the data was transferred from BaseClear to WBVR for further analysis.

### Data analysis and statistics

**Fig. 1:**
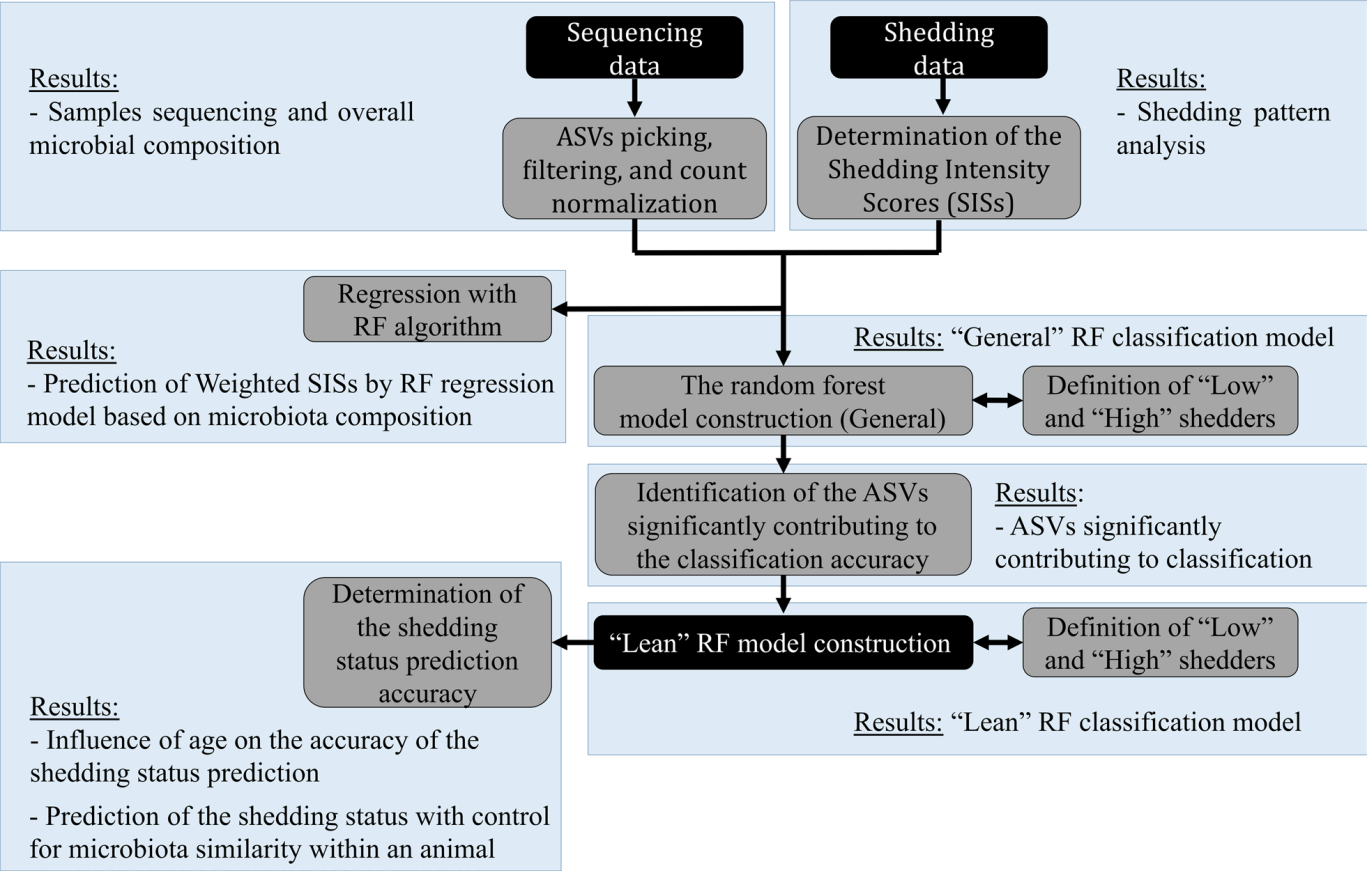
Overview of the data analysis workflow. Black boxes indicate input data and the final model; grey boxes correspond to data processing steps and arrows show connection between them. Names of the grey boxes are identical to the titles of data analysis subsection in the Material and Methods section. Blue rectangles show correspondence between data analysis steps and the Results section

A schematic outline of the data analysis steps is shown in Fig. 1. The outline shows the major steps of analysis, corresponding results and connections between them. Subheadings of the Data analysis subsections are identical to the names of the grey coloured action boxes in Fig. 1.

The complete data analysis pipeline is available as a GitHub project at https://github.com/AlexanderUm/WBVR_MAP_Microbiota. Additional file 2 contains the samples’ metadata used for the data processing.

### Determination of the shedding intensity scores

Isolation and quantification of the MAP colonies were performed according to methods published previously [25, 26]. Based on the number of colonies per tube a Shedding Intensity Score (SIS) was assigned to each sample. The SISs were assigned as following: 0—no colonies observed; 1—from 1 to 10 colonies observed; 2—from 11 to 100 colonies observed; 3—more than 100 colonies observed (Additional file 3). The SIS values reflect intensity of MAP shedding at a given time point and were used for further calculations of shedding scores. Based on the individual SIS a Weighted SIS was calculated. For calculation of Weighted SISs the sum of all SISs per animal was divided by the number of samples from that animal. The Weighted SISs were taken as an objective indication of MAP shedding severity. Weighted SISs were calculated for all available samples (I) and for samples from the first 20 months of the experiment (II). The Weighted SISs II were calculated with the goal to mitigate effects of differences in the time an animal was present in the experiment (Additional file 4: Fig. S1A). In total 20 animals were enrolled in the experiment, however, some were culled before the end of the experiment. In total, eight animals were culled between 20 and 26 months of age (“Early Culled”) due to the inability to breed or health related problems. Weighted SISs I and Weighted SISs II in the subset of animals which survived until the end of the experiment showed a high and significant correlation (Pearson; r = 0.89, *p* < 0.0001), and further analysis were performed using Weighted SISs II (Additional file 4: Fig. S1B).

### ASVs picking, filtering, and count normalization

The raw data was processed following the DADA2 v1.14.1 pipeline [29] in R v3.6.2 statistical and programming environment [30]. The final output of DADA2 pipeline is an abundance table of amplicon sequence variants (ASVs). An ASV is a unique sequence of a marker gene that approximates a microbial species or strain and could be used as the lowest taxonomic units (for more details see publication of Callahan et al. [26]). In short, primers and low quality bases were truncated from the reads, the model of error-rate within sequences was inferred from the data, ASVs were picked and chimeric sequences removed. AVSs were taxonomy assigned as implemented in DADA2 (function “assignTaxonomy”) using the suggested version of the manually curated Silva v138 database [31]. Taxonomic and abundance tables produced by the DADA2 pipeline were combined with metadata into a phyloseq object [32]. Prior to further analysis ASVs were filtered to retain only taxa that were present in more than three samples. Also, samples with less than 1000 reads were removed. Sufficiency of the sequencing depth was assessed by rarefaction curves with a 200 bp step as implemented in the package vegan (v 2.5–6). Prior to further analysis, ASV count data was normalized using cumulative sum scale (CSS) transformation [33].

### The random forest model construction (General)

The random forest (RF) algorithm [34] as implemented in the randomForest v4.6–14 package [35] was used for sample classification and regression. The RF model was fine-tuned by determining the optimal number of taxa at each split (“mtry” parameter) and the number of classification trees (“ntree” parameter). The “mtry” parameter was tuned using the tuneRF function (randomForest package) with step factor two. The optimal number of classification trees was determined by constructing the RF model five times with a different number of trees, and subsequent identification of the models with the lowest out of bag error (OOBE).

### Definition of “Low” and “High” shedders

The Weighted SISs were used as a guide to assign animals as a “Low” or “High” shedder, however no obvious split between animals was observed. Therefore, animals were classified into “Low” or “High” shedders based on accuracy of RF classification model. Several RF models with various configurations of “High” and “Low” shedding groups were constructed. Samples were assigned as “High” shedders if their Weighted SIS II was above the selected cut-off value and as “Low” shedders if below. Cut-off values with a range between between 0.45 and 1.19 were used to capture shifts in the shedding intensity and a RF model was built for each available Weighted SIS II within this range.

### Regression with RF algorithm

The regression model was built as implemented in the randomForest package. The sufficient number of trees for the RF model was identified as follows. A RF model was built using default parameters (randomForest) and an excessively large number of trees (20,000). The built model was used for plotting relationships between accuracy and the number of used trees (plot(RF)). A total of 7501 trees were used as the final ntree parameter for the model with the default mtry parameter.

To test the prediction accuracy beyond OOBE a subtraction approach was used as described below. Consequentially several RF regression models were built, each time using a data-set with ten uniquely subtracted samples until no possibility for unique subtraction was left. RF regression models were used to predict values of the corresponding subtracted values (generic R function “predict”). To account for the random variations in RF models the model building for each set of subtracted samples was repeated five times.

### Identification of the taxa significantly contributing to the classification accuracy and “Lean” RF model construction

Taxonomic features with a significant contribution to the RF accuracy were identified by a permutation test as implemented in rfPermute v2.1.81 package [36]. Only features with the significance levels below threshold values (alpha = 0.05) for the mean decrease accuracy and the mean decrease purity (Gini purity index [35]) were considered as important for classification. Significant ASVs were used as input features for building a new simplified RF model (“Lean”). The “mtry” and “ntree” parameters for the simplified RF model were optimised as described above.

### Determination of the shedding status prediction accuracy

Accuracy of the shedding status prediction was assessed by construction of the receiver operating characteristic (ROC) curve and calculation of the area under the curve (AUC). To calculate ROC curve and AUC a dataset was split into “training” and “validation” datasets. The “training” dataset was used to construct the RF classification model (package *randomForest*) and shedding status of samples from the corresponding “validation” dataset was predicted (generic R function “predict”). The resulting prediction was used to construct ROC curves and calculated AUC (package *ROCR*).

To understand the influence of cows' age on the accuracy of classification the ROC curve and AUC for different age groups were calculated. Three age groups were defined: Early (n = 82)—before 12 months of age, Middle (n = 90)—12 to 24 months of age and Late (n = 85)—older than 24 months of age. The ROC curve and AUC for a specific age group was calculated based on the group specific “validation” and “training” datasets. Fifty percent of the samples from the target age group was randomly drawn to create a “validation” dataset. The samples from the other age groups and not drawn samples from the target group comprised a “training” dataset. The overall ROC curve and AUC were estimated using 20% of randomly drawn data points from all samples as a “validation” dataset and the rest of the samples as a “training” dataset. Splitting of the target data into “validation” and “training” with consequent evaluation of the ROC curve and AUC was repeated 99 times for each age group and the complete dataset.

Next, we ruled out the possibility of samples classification into a shedding group due to similarity of microbiota within one animal during its lifespan. We used all samples from a single animal as the “validation” dataset and the rest of the samples as the “training” dataset. This procedure was repeated for every animal and the results of prediction were compared with the shedding status.

### Data wrangling and visualization

For data wrangling and visualization, the package tidyverse v1.2.1 [37] was used. For visualization of heatmaps the package ComplexHeatmaps [38] was used.

## Results

### Shedding pattern analysis

**Fig. 2:**
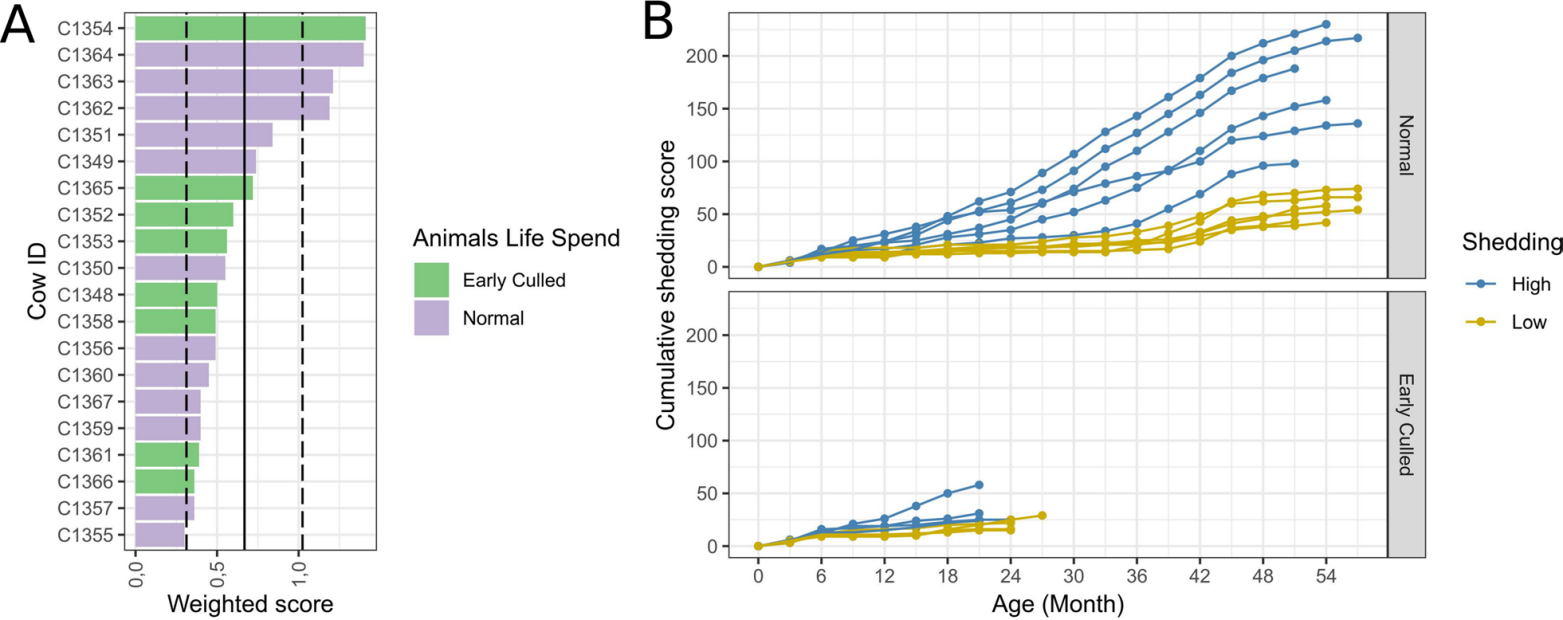
**A** Bar chart of Weighted shedding intensity scores (SIS). The solid blue line marked the average weighted SIS and dashed lines marked 0.5 of Standard deviation. **B** Line/scatter plot of cumulative shedding values. Each dot represents the sum of shedding scores prior and including this time point. Lines and dots are coloured by shedding status that was assigned based on optimal separation by RF classification model (See “Results”: “General” RF classification model)

No apparent division in “Low” and “High” shedders among animals based on the Weighted SISs was observed. A gradual increase in Weighted SISs from 0.3 (lowest observed) to 0.84, slight jump from 0.84 to 1.19 and further gradual increase to 1.41 (highest observed) was observed. The mean shedding value among all samples was 0.53 (Fig. 2A, B).

### Samples sequencing and overall microbial composition

Two hundred fifty seven samples remained in the sample-set after the quality control with a median of 12 samples per cow, a maximum of 22, and a minimum of 8 (Additional file 4: Table S1). In total, 3,973,061 reads passed the initial quality control and filtering as described in the material and methods section. The median value of reads per sample was 14,000 reads with a minimum of 1415 and a maximum of 44,407 (Additional file 4: Fig. S2A). The rarefaction curves showed sufficient sequencing depth to capture microbial diversity at ASV level (Additional file 4: Fig. S2 B).

Overall 4940 ASVs from 15 microbial phyla remained in the dataset after the filtering steps. However, only *Firmicutes* (from 61.3 to 64.3%, 95% CI), *Bacteroidota* (from 29.2 to 32.2%, 95% CI), *Verrucomicrobiota* (from 2.0 to 2.7%, 95% CI), *Actinobacteriota* (from 1.3 to 1.7%, 95% CI) received averagely more than 1% of the reads across all samples (Additional file 4: Fig. S3, Table S2).

### Prediction of weighted SISs by RF regression model based on microbiota composition

The absolute differences between predicted and actual Weighted SISs ranged from 0.0007 up to 0.81, with the median value of 0.2. A significant correlation between animal ID and absolute difference between predicted and actual Weighted SISs (Pearson; r =  − 0.85, *p* < 0.0001) was found, but not with the age of the animals. Overall averages of the predicted Weighted SISs were significantly correlated with the actual Weighted SISs (Pearson; r = 0.83, *p* < 0.0001), however, accuracy of the prediction varied strongly per individual sample and per animal (Additional file 4: Fig. S4).

### “General” RF classification model

**Fig. 3:**
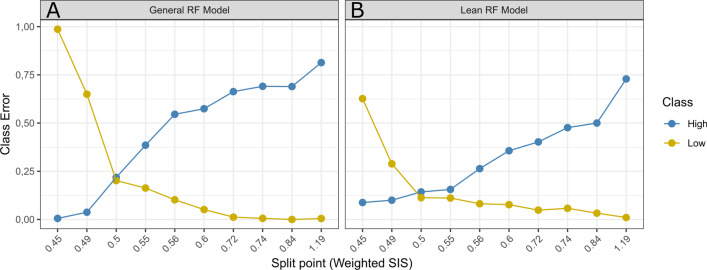
Scatter plot of class errors correspond to General (**A**) and Lean (**B**) random forest (RF) models with a different composition of “High” and “Low” shedding groups. General RF model built with all ASVs passed quality control and Lean RF model employed only AVS significantly contributing to classification. On the x-axis Weighted Shedding Intensity Scores (SISs) are shown that were used as a split point for the groups division, and y-axis depict the class error expressed in proportion

The best prediction outcome for the RF model based on all ASVs (“General” RF) was observed when samples with the Weighted SISs ≤ 0.5 were assigned as “Low” shedders and all above assigned as “High”. At that point, class errors for the “Low” and “High” shedders were comparable with each other (Fig. 3).

A larger number of trees (ntree = 15,001) resulted in a more stable classification model, and the accuracy of classification increased when a larger than the default number of features (ASVs) per split (mtry = 568; Additional file 4: Fig. S5) was used. The final classification model resulted in an OOBE of 22.2% and class errors of 28.7% and 16.3% for “High” and “Low” shedding groups respectively.

### ASVs significantly contributing to classification

In total, 192 ASVs were identified as significantly contributing to the accuracy of classification (Additional file 5). From the phylogenetic point of view, identified ASVs were distributed among nine different phyla, 12 classes, 25 orders, 41 families and 54 genera.

**Fig. 4:**
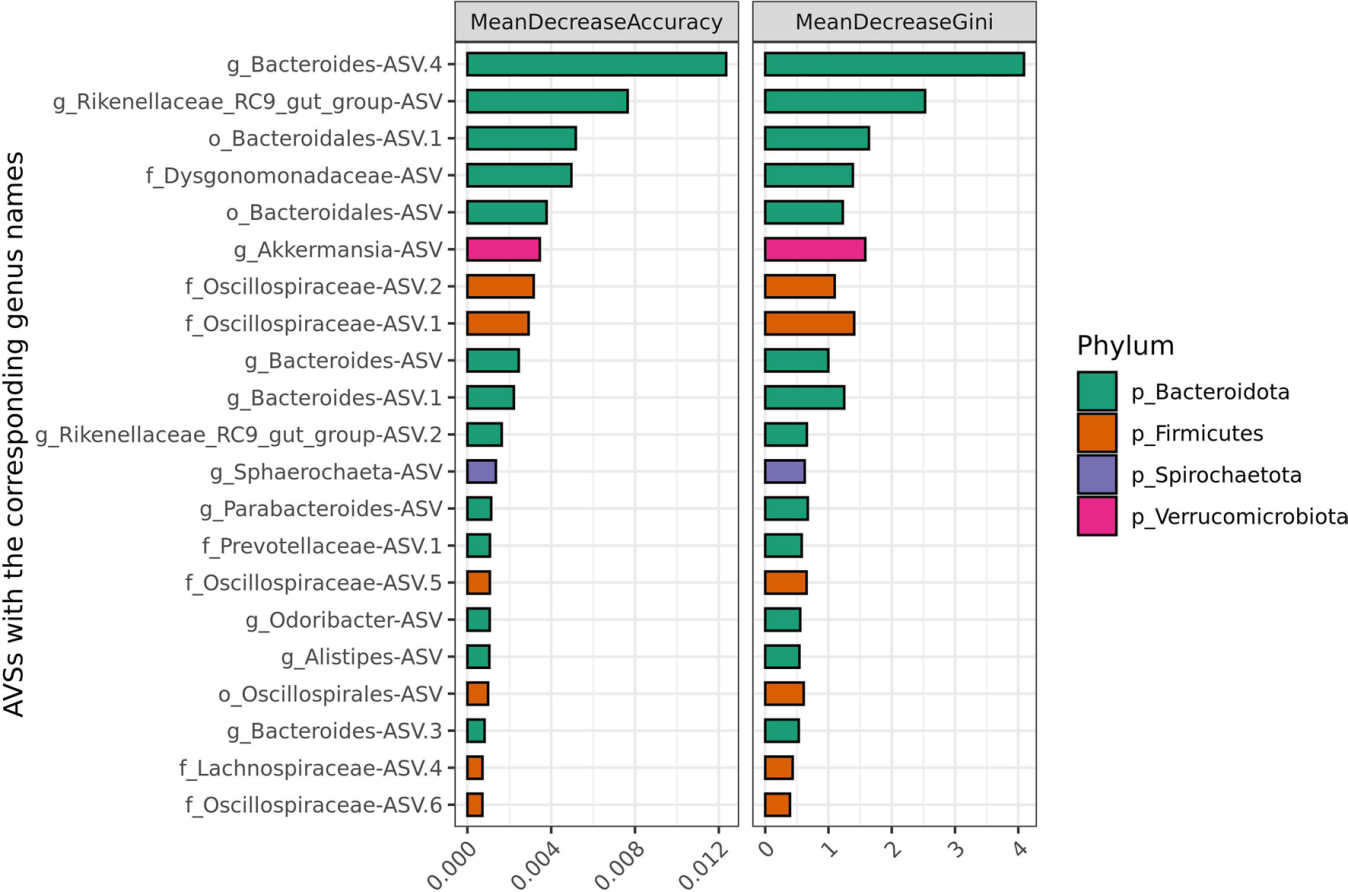
Top 21 ASVs with the highest contribution to Random Forest classification accuracy. ASVs named by a corresponding genus, or lowest available taxonomic classifier as indicated by prefix letter

The majority of ASVs that contributed to classification accuracy belonged to *Firmicutes* (108 ASVs) and *Bacteroidota* (58 ASVs) phyla followed by *Verrucomicrobiota* (10 ASVs) and *Actinobacteriota* (7 ASVs), and the remaining phyla were represented only by 1–3 ASVs. The overall composition of the significantly contributing taxa mirrored the overall microbiota composition, however, out of the top 21 ASVs contributing to the Accuracy of the RF classification model, more ASVs belonged to *Bacteroidota* (13 ASVs) than to *Firmicutes* (6 ASVs; Fig. 4).

**Fig. 5:**
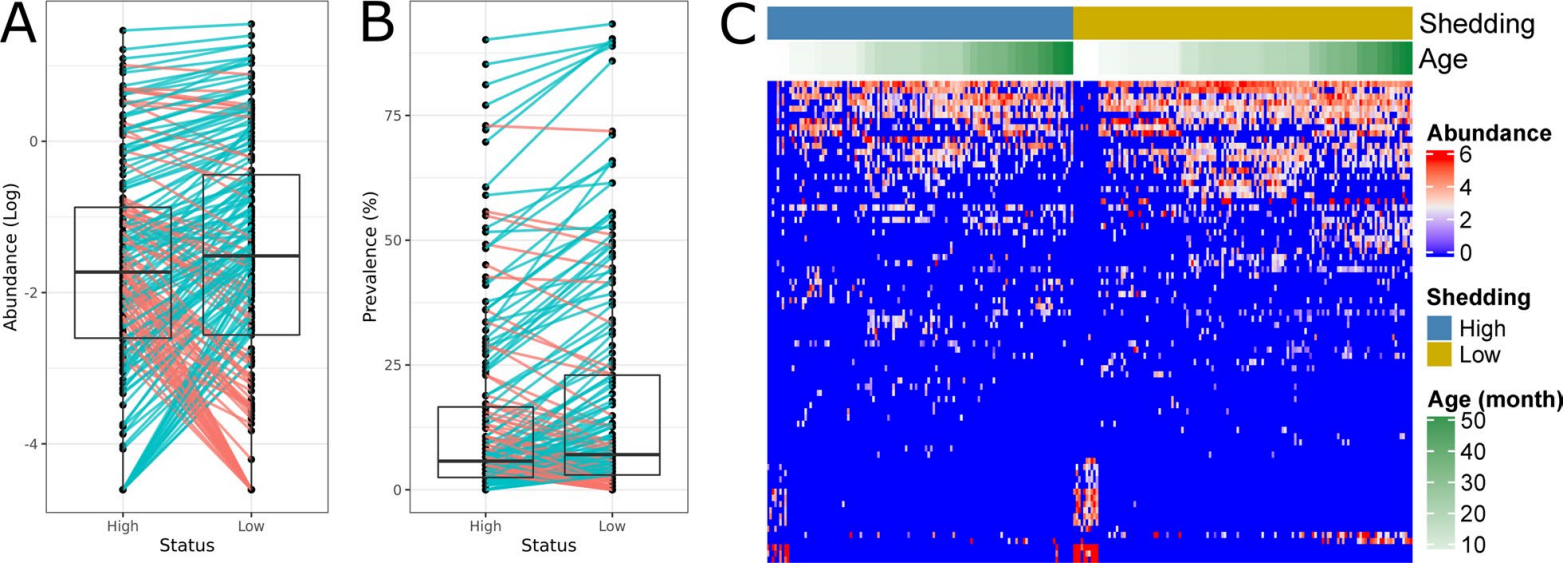
ASVs significantly contributed to Random Forest model classification accuracy. The boxplots show differences in log transformed averaged abundance (**A**) and average prevalence (**B**); green (higher in “Low” group) and red (higher in “High” group) lines represent differences in abundance or prevalence of individual ASVs. **C** Heatmap shows CSS normalized abundance of the ASVs, with samples arranged in columns and ASVs in rows

The differences in the average CSS normalized abundance and the prevalence of ASVs contributed to the classification accuracy between “High” and “Low” shedding groups ranged from 0.01 to 1.59 and from 0.5 to 34% respectively. Overall, no large difference in ASVs prevalence, nor abundance in the “Low” versus “High” shedding groups (Fig. 5A, B) was observed. However, a clear distinction in ASVs identified as significantly contributing to classification was observed between samples from the animals with the milk (pre-weaning) and solid diet (post-weaning; Fig. 5C).

### “Lean” RF classification model

The RF classification model was built using only ASVs that were identified as significantly contributing to classification (“Lean” RF classification model) with the goal to reduce the number of input features, decrease computational time and subsequently improve the accuracy of classification. Similarly to the “General” RF model, the best split for the “Lean” RF model was observed when samples with the Weighted SIS ≤ 0.5 were assigned to the “Low” shedding group, and all above to the “High” shedding group. However, the “Lean” RF model showed a similar classification accuracy for the Weighted SIS split point 0.5 and 0.55, rather than a single best split point as it was observed for the General RF model (Fig. 3).

The “Lean” RF model showed the best accuracy of classification when 28 ASVs per split (mtry parameter) and an excessively sufficient number of trees (ntree = 15,001) were used. The “Lean” RF model showed higher accuracy than the “General” RF model and resulted in 12.84% OOBE. The class errors were 9.63% for the “Low” and 16.4% for the “High” shedding groups.

### Influence of age on the accuracy of the shedding status prediction

**Fig. 6:**
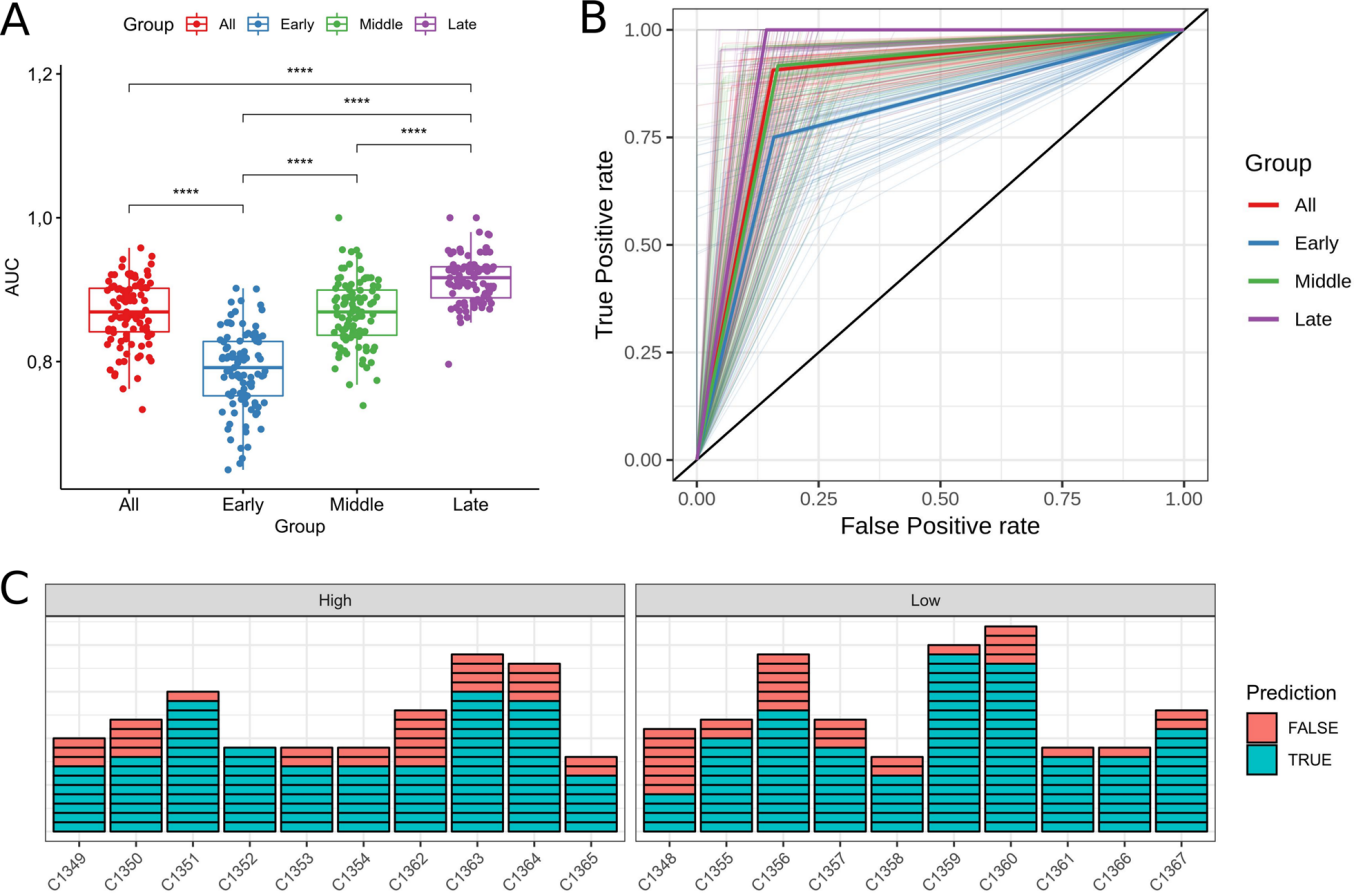
Accuracy of the samples classification into shedding groups. **A** and **B** show classification for samples from the different life periods: “Early”—younger than 12 month, “Middle”**—**from 12 to 24 month, “Late”—older than 24 months, and “All”—samples randomly drawn from all age groups. **A** Box-plot of AUC per life period group—every dot represents AUC obtained in a single permutation; **** level of significance corresponds to *p* < 0.0001. **B** ROC curves plot—thin half-transparent lines represent ROC curves of individual permutations and thick solid lines are median ROC curves per group. **C** shows the number of miss-classified samples per animal when all samples from the corresponding animal removed from the RF model training dataset and used as the validation; samples are faceted by shedding status

A progressive improvement in the accuracy of the shedding status prediction from the “Early” to “Late” age groups was observed (Fig. 6A, B). The highest AUC was observed for the samples from the animals older than 24 months (“Late” group; AUC from 0.91 to 0.92, 95% CI) and the lowest in the samples from the animals younger than 12 months (“Early” group; AUC from 0.78 to 0.79, 95% CI). Prediction for samples from animals between 12 and 24 month of age (“Middle” group) showed an intermediate accuracy (AUC from 0.86 to 0.87, 95% CI) that was not significantly different from the accuracy observed for the complete dataset (“All” group; AUC from 0.86 to 0.87, 95% CI). In addition, a higher consistency in observer AUC between permutations was observed in the “Late” (SD = 0.036) group in comparison with “Middle” (SD = 0.046) and “Early” (SD = 0.054) groups.

### Prediction of the shedding status controlled for microbiota similarity within an animal

The number of correctly classified samples varied per animal (Fig. 6C) with 95% CI between 70.3% and 83.7%. However, only 5 out of 11 samples from cow C1348 were classified correctly. The overall AUC with this classification approach was 0.77. No clear age dependent pattern for misclassification of the samples was observed (Additional file 4: Fig. S6).

## Discussion

To our knowledge, this is the first study that leveraged relationships between the gut microbiota composition and MAP infection in cows to predict severity of MAP shedding in the environment. Effective diagnosis of contagious diseases is a cornerstone when it comes to their control and prevention. However, a positive test does not always provide guidance for further action, where a forecasting model could give a direction for disease control options and mitigation of financial losses. It is particularly relevant for diseases with a slow progressive development and a variable outcome such as Johne’s disease.

Our prediction model in the best-case scenario showed an AUC from 0.86 to 0.87 (95% CI) for separation of the “Low” from the “High” shedding cows. It is difficult to compare these results with the conventional diagnostic methods based on culturing, PCR or ELISA. The conventional MAP diagnostic methods are designed to give the information about presence or absence of the pathogen at the time of sampling rather than predict development of the disease and amount of the MAP shed by the infected cow during its lifetime.

Accuracy of our model was age sensitive and showed improvement from young to the old age groups. Similar observations have been made for conventional diagnostic methods. The specificity of culture and ELISA based methods has been shown to be greatly improved in older cows with more advanced stages of the disease [39, 40]. The slow-progressive nature of MAP infection, especially the slow development of lesions as well as MAP specific immune responses is the most likely to be the explanation for these observations. Improved sensitivity of the microbiota based model could be attributed to extensive pathogen-induced changes in the intestinal environment and the microbiome, where the sensitivity of culturing or PCR based methods solely relies on the increase of MAP replication and faecal shedding. This difference is important, since decoupling of a predictive or diagnostic method from the presence of the pathogen in a sample could greatly improve robustness of the method. MAP cells tend to clump together rather than grow as a planktonic culture, which inherently creates an uneven distribution of MAP in faecal samples. The heterogeneous distribution of MAP in biological matrices can seriously compromise sensitivity of tests that are reliant on the presence of MAP in a small tested sample. Currently, only immunological tests (predominantly absorbed ELISA tests) are decoupled from the direct detection of MAP in samples. However, immunological tests also struggle with ambiguous results due to arbitrary defined cut-off values between positive and negative samples.

Detection of MAP in early stages of the disease with classical methods is particularly difficult due to intermittent or lack of pathogen shedding [41] as well as slow development of the adaptive immune response [42]. The results of the current study however show satisfactory accuracy (AUC from 0.78 to 0.79, 95% CI) of classification into “Low” and “High” shedding groups even at an early stage of infection. Based on these results it is tempting to speculate about the possibility of microbiota composition based diagnostic tests that will be superior in detection of the paratuberculosis at the very early stages of the disease. However, that will require an experiment with a different study design that will allow the comparison between a large number of healthy and cows infected with MAP.

The microbiota based shedding intensity classifier was built using the RF algorithm. The RF shown to be a simple, effective and “white box” machine learning approach that can handle various data types [14], including microbiota compositional data [43, 44]. Flexibility of the approach allowed construction of an accurate classifier, but also identification of the MAP infection relevant ASVs biomarkers and test possibilities.

Construction of the “General” RF model allowed the identification of ASVs for the final (“Lean”) classification model. When comparing performance of the “General” and the “Lean” RF model a clear improvement in accuracy of classification as well as a reduction of the time required for the model training was observed. The selection of features is commonly used prior to application of a machine learning algorithm [45] to reduce the amount of noise from irrelevant data, and overcome the curse of dimensionality [46].

The RF regression model that was developed showed a moderate performance. Despite a statistically significant correlation between predicted and actual Weighted SISs the accuracy was not satisfactory. In particular, the samples for the animals with lower Weighted SISs had a large discrepancy between the actual and predicted values. Nevertheless, a regression model to predict shedding of MAP could be a useful tool in certain situations, but the model construction will require a larger dataset and a different experimental design.

Optimization of RF parameters was an important step to improve accuracy of the classification model. This sensitivity of RF to changes in *mtry* parameter was shown previously in application to the gene expression data [47].

Definition of a response variable and groups for classification is a crucially important step in development of a the classification model. In our case, the shedding intensity data was inferred from the number of cultured MAP colonies and was used as the response variable. The scores of the shedding intensity rather than actual count of the cultivated colonies were used to allow a higher level of generalization, which could be beneficial for smaller data-sets. Nevertheless, the actual count of cultivated colonies or qPCR assessed gene copy number could be useful for larger datasets, and could allow for inference of the shedding values rather than classification into shedding intensity groups. However, due to high variability of microbiota composition between individual animals this approach could also suffer from a low precision with less interpretable results.

When it comes to the definition of the groups for classification—there is no clear guide what could be considered low or high shedding cow, and cut-off is usually set arbitrarily. A gradual increase in the Weighted SISs in studied animals was observed without a clear separation into “High” or “Low” shedders. MAP infected animals often show intermediate shedding where periods of severe shedding are followed by low or completely shedding free periods [41, 48]. Therefore, when samples are not collected longitudinally the division into high and low shedders could be more prominent [49]. However, differences became more subtle when shedding score was evaluated during the lifetime of the animal. An optimal separation into “Low” and “High” shedders was obtained by comparing the accuracy of RF models built with the different configurations of the groups. This approach is suitable for the “proof of concept” research, such as presented in this paper, however, the development of a real world applicable MAP shedding prediction tools will require a larger number of animals with a clearer division into shedding groups.

One of the great advantages of the RF classification algorithm is the possibility to get insight in the contribution of individual features to classification. In total, 4940 ASVs were identified across all samples, nevertheless, only 4% from the total number was important for the classification. This is not surprising since not every microbial taxa will respond to MAP infection, or have protective qualities against it. In addition, microbiota composition has been shown to vary between individuals and only a relatively small number of taxa comprises a shared core [50, 51]. The current dataset consisted from samples collected in a longitudinal experiment that captured all stages of animal development with the consequent age and season related microbiota variations, which further decreased the number of ASVs that could have a meaningful impact on the classification. In the current study, age related change in diet from milk to solid food and following overall microbial composition are reflected in differences in ASVs significant to classification. This is an expected finding, nevertheless, it underlines the importance of an experimental design adequate to reach the intended goals. In our case, the number of samples available from the animals of early age (milk diet) was a small proportion of total samples and did not allow for a separate investigation.

Among the top 20 ASVs contributing to classification, the majority had a significantly higher abundance in the “Low” (18 ASVs) than in the “High” (2 ASVs) shedding group. It is difficult to speculate about the function of ASVs and why they are more abundant in the “Low” shedding group, due to the limited taxonomic resolution of the microbiota profiling based on an amplified 16S rRNA fragment [52]. In addition, almost half (8 out of 20) ASVs were not assigned even to a genus level, increasing uncertainty regarding the role of a microbial feature. Future research may benefit from advances in sequence technologies and strategies such as shotgun metagenomic sequencing. Nevertheless, even a high-level taxonomic assignment can give an indication of the ecological niche, albeit with limitations. ASVs from genus *Bacteroides* showed the highest contribution to the accuracy of classification. Four out of the top 20 ASVs belonged to genus *Bacteroides*, and all of them have significantly higher abundance in the “Low” in comparison with the “High” shedding group. Members of the genus *Bacteroides* can be found only as a part of the mammalian gastrointestinal microbiota [53]. They are generally regarded as beneficial commensals when they resign in their niche, however, some strains are pathogenic and can cause severe infections [54]. Higher abundance of *Bacteroides* in the “Low” shedding group could be an indication of their protective properties and competitive exclusion of MAP, however further research will be needed. Intriguingly, an ASV from the genus *Akkermansia* had a significantly higher abundance in the “High” shedding group. *Akkermansia* is a mucin degrading commensal bacteria with a tight connection with the host [55]. It is generally considered as a beneficial microbe in humans [56]. The higher abundance in the “High” shedding group is puzzling, but could be explained by physiological changes in the mucosal layer due to MAP colonization and as a consequence an expansion of the *Akkermansia* habitat. However, the possibility of a more direct relationship between MAP and *Akkermansia* should not be discarded.

## Conclusions

In this proof-of-concept-study, showed evidence that future shedding severity of MAP by cows can potentially be forecast based on the composition of the intestinal microbiota. To our knowledge this is the first approach that showed a potential to predict severity of MAP infection at early stages of the infection. Forecasting of MAP infection progression will help to develop management strategies in farms with the high prevalence, endemic and or hard to control MAP infection as it will provide farmers and veterinarians with a tool to select animals for culling. An indiscriminate culling based on MAP specific test results indicating presence or absence of MAP may be an economically non-viable strategy for areas with endemic MAP. Therefore, microbiota based prediction of the infection development can help mitigate the economic burden of MAP and limit spread of the infection in populations where MAP is endemic by preferentially targeting animals with a likely higher lifetime contribution to MAP transmission and environmental contamination.

## Supplementary Information

**Additional file 1:** Sample selection data.

**Additional file 2:** Sample metadata.

**Additional file 3:** Fecal shedding intensity data.

**Additional file 4:** Shedding intensity score calculation.

**Additional file 5:** Significantly contributing ASV.

## Data Availability

The raw sequencing data generated during the current study are available in the NCBI repository under BioProject No. PRJNA706519. Data will be accessible upon publication. Metadata provided as an additional file with the manuscript. Full data analysis pipeline is available as a GitHub repository via the link: https://github.com/AlexanderUm/WBVR_MAP_Microbiota.

## Abbreviations

MAP: *Mycobacterium Avium* Subsp. *Paratuberculosis*
RF: Random forest
OOBE: Out of bag error
ASV: Amplicon sequence variant
PCR: Polymerase chain reaction
ELISA: Enzyme-linked immunosorbent assay
AUC: Area under the curve
SIS: Shedding Intensity Score
CI: Confidence interval
ROC: Receiver operating characteristic
CSS: Cumulative sum scaling

## References

[1] Cocito C, Gilot P, Coene M, de Kesel M, Poupart P, Vannuffel P (1994). Paratuberculosis. Clin Microbiol Rev.

[2] Sweeney RW (1996). Transmission of paratuberculosis. Vet Clin N Am Food Anim Pract.

[3] Taniguchi Y, Sakakibara S-I, Fujihara M, Yagi A, Fujiyoshi S (2020). The association between detection of *Mycobacterium avium* subsp. *paratuberculosis* DNA in feces and histopathological classification. J Vet Med Sci.

[4] Biemans F, Ben Romdhane R, Gontier P, Fourichon C, Ramsbottom G, More SJ (2021). Modelling transmission and control of *Mycobacterium avium* subspecies *paratuberculosis* within Irish dairy herds with compact spring calving. Prev Vet Med.

[5] Whitlock RH, Wells SJ, Sweeney RW, Van Tiem J (2000). ELISA and fecal culture for paratuberculosis (Johne’s disease): sensitivity and specificity of each method. Vet Microbiol.

[6] Nielsen SS, Grønbæk C, Agger JF, Houe H (2002). Maximum-likelihood estimation of sensitivity and specificity of ELISAs and faecal culture for diagnosis of paratuberculosis. Prev Vet Med.

[7] Koets AP, Eda S, Sreevatsan S (2015). The within host dynamics of *Mycobacterium avium* ssp. *paratuberculosis* infection in cattle: where time and place matter. Vet Res.

[8] Bannantine JP, Bermudez LE (2013). No holes barred: invasion of the intestinal mucosa by *Mycobacterium avium* subsp. *paratuberculosis*. Infect Immun.

[9] Derakhshani H, De Buck J, Mortier R, Barkema HW, Krause DO, Khafipour E (2016). The features of fecal and ileal mucosa-associated microbiota in dairy calves during early infection with *Mycobacterium avium* Subspecies *paratuberculosis*. Front Microbiol.

[10] Fecteau M-E, Pitta DW, Vecchiarelli B, Indugu N, Kumar S, Gallagher SC (2016). Dysbiosis of the fecal microbiota in cattle infected with *Mycobacterium avium* subsp *paratuberculosis*. PLoS ONE.

[11] Arrazuria R, Elguezabal N, Juste RA, Derakhshani H, Khafipour E (2016). *Mycobacterium avium* Subspecies *paratuberculosis* infection modifies gut microbiota under different dietary conditions in a rabbit model. Front Microbiol.

[12] Adlung L, Elinav E, Greten TF, Korangy F (2020). Microbiome genomics for cancer prediction. Nat Cancer.

[13] Liu Y, Meric G, Havulinna AS, Teo SM, Ruuskanen M, Sanders J (2020). Early prediction of liver disease using conventional risk factors and gut microbiome-augmented gradient boosting. medRxiv.

[14] Olivares M, Walker AW, Capilla A, Benítez-Páez A, Palau F, Parkhill J (2018). Gut microbiota trajectory in early life may predict development of celiac disease. Microbiome.

[15] Zhou Y, Xu ZZ, He Y, Yang Y, Liu L, Lin Q (2018). Gut microbiota offers universal biomarkers across ethnicity in inflammatory bowel disease diagnosis and infliximab response prediction. MSystems..

[16] Kalliomäki M, Carmen Collado M, Salminen S, Isolauri E (2008). Early differences in fecal microbiota composition in children may predict overweight. Am J Clin Nutr.

[17] Faith JJ, Guruge JL, Charbonneau M, Subramanian S, Seedorf H, Goodman AL (2013). The long-term stability of the human gut microbiota. Science.

[18] Huang S, Ji S, Wang F, Huang J, Alugongo GM, Li S (2020). Dynamic changes of the fecal bacterial community in dairy cows during early lactation. AMB Express.

[19] Tang MT, Han H, Yu Z, Tsuruta T, Nishino N (2017). Variability, stability, and resilience of fecal microbiota in dairy cows fed whole crop corn silage. Appl Microbiol Biotechnol.

[20] Zhang Q, Li C, Niu X, Zhang Z, Li F, Li F (2019). The effects of milk replacer allowance and weaning age on the performance, nutrients digestibility, and ruminal microbiota communities of lambs. Anim Feed Sci Technol.

[21] Odamaki T, Kato K, Sugahara H, Hashikura N, Takahashi S, Xiao J-Z (2016). Age-related changes in gut microbiota composition from newborn to centenarian: a cross-sectional study. BMC Microbiol.

[22] Midani FS, Weil AA, Chowdhury F, Begum YA, Khan AI, Debela MD (2018). Human gut microbiota predicts susceptibility to vibrio cholerae infection. J Infect Dis.

[23] Shalev-Shwartz S, Ben-David S (2014). Understanding machine learning: from theory to algorithms.

[24] Marcos-Zambrano LJ, Karaduzovic-Hadziabdic K, Loncar Turukalo T, Przymus P, Trajkovik V, Aasmets O (2021). Applications of machine learning in human microbiome studies: a review on feature selection, biomarker identification, disease prediction and treatment. Front Microbiol.

[25] Langelaar MFM (2005). Heat shock protein 70 and bovine paratuberculosis.

[26] Ganusov VV, Klinkenberg D, Bakker D, Koets AP (2015). Evaluating contribution of the cellular and humoral immune responses to the control of shedding of *Mycobacterium avium* spp. *paratuberculosis* in cattle. Vet Res.

[27] Vary PH, Andersen PR, Green E, Hermon-Taylor J, McFadden JJ (1990). Use of highly specific DNA probes and the polymerase chain reaction to detect *Mycobacterium paratuberculosis* in Johne disease. J Clin Microbiol.

[28] Herlemann DPR, Labrenz M, Jürgens K, Bertilsson S, Waniek JJ, Andersson AF (2011). Transitions in bacterial communities along the 2000 km salinity gradient of the Baltic Sea. ISME J.

[29] Callahan BJ, McMurdie PJ, Rosen MJ, Han AW, Johnson AJA, Holmes SP (2016). DADA2: high-resolution sample inference from Illumina amplicon data. Nat Methods.

[30] Team RC. R: a language and environment for statistical computing. Vienna: R Foundation for Statistical Computing; 2017.

[31] Quast C, Pruesse E, Yilmaz P, Gerken J, Schweer T, Yarza P (2013). The SILVA ribosomal RNA gene database project: improved data processing and web-based tools. Nucleic Acids Res.

[32] McMurdie PJ, Holmes S (2013). phyloseq: an R package for reproducible interactive analysis and graphics of microbiome census data. PLoS ONE.

[33] Paulson JN, Stine OC, Bravo HC, Pop M (2013). Differential abundance analysis for microbial marker-gene surveys. Nat Methods.

[34] Breiman L (2001). Random forests. Mach Learn.

[35] Liaw A, Wiener M. Classification and regression by RandomForest. Forest. 2001;23.

[36] Archer E. rfPermute: estimate permutation p-values for Random Forest importance metrics. R package version. 2016;1(2).

[37] Wickham H. The tidyverse. R package ver. 2017;1(1).

[38] Gu Z, Eils R, Schlesner M (2016). Complex heatmaps reveal patterns and correlations in multidimensional genomic data. Bioinformatics.

[39] Nielsen SS, Toft N (2006). Age-specific characteristics of ELISA and fecal culture for purpose-specific testing for paratuberculosis. J Dairy Sci.

[40] Blanco Vázquez C, Alonso-Hearn M, Juste RA, Canive M, Iglesias T, Iglesias N (2020). Detection of latent forms of *Mycobacterium avium* subsp. *paratuberculosis* infection using host biomarker-based ELISAs greatly improves paratuberculosis diagnostic sensitivity. PLoS ONE.

[41] Mortier RAR, Barkema HW, Orsel K, Wolf R, De Buck J (2014). Shedding patterns of dairy calves experimentally infected with *Mycobacterium avium* subspecies *paratuberculosis*. Vet Res.

[42] Huda A, Jungersen G, Lind P (2004). Longitudinal study of interferon-gamma, serum antibody and milk antibody responses in cattle infected with *Mycobacterium avium* subsp. *paratuberculosis*. Vet Microbiol.

[43] Belk A, Xu ZZ, Carter DO, Lynne A, Bucheli S, Knight R (2018). Microbiome data accurately predicts the postmortem interval using random forest regression models. Genes.

[44] Saulnier DM, Riehle K, Mistretta TA, Diaz MA, Mandal D, Raza S (2011). Gastrointestinal microbiome signatures of pediatric patients with irritable bowel syndrome. Gastroenterology.

[45] Blum AL, Langley P (1997). Selection of relevant features and examples in machine learning. Artif Intell.

[46] Qi Y. Random forest for bioinformatics. In: Ensemble machine learning. Springer; 2012. p. 307–23.

[47] Statnikov A, Wang L, Aliferis CF (2008). A comprehensive comparison of random forests and support vector machines for microarray-based cancer classification. BMC Bioinform.

[48] Raizman EA, Fetrow J, Wells SJ, Godden SM, Oakes MJ, Vazquez G (2007). The association between *Mycobacterium avium* subsp. *paratuberculosis* fecal shedding or clinical Johne's disease and lactation performance on two Minnesota, USA dairy farms. Prevent Vet Med..

[49] Crossley BM, Zagmutt-Vergara FJ, Fyock TL, Whitlock RH, Gardner IA (2005). Fecal shedding of *Mycobacterium avium* subsp. *paratuberculosis* by dairy cows. Vet Microbiol.

[50] Durso LM, Harhay GP, Smith TPL, Bono JL, DeSantis TZ, Harhay DM (2010). Animal-to-animal variation in fecal microbial diversity among beef cattle. Appl Environ Microbiol.

[51] Falony G, Joossens M, Vieira-Silva S, Wang J, Darzi Y, Faust K (2016). Population-level analysis of gut microbiome variation. Science.

[52] Fuks G, Elgart M, Amir A, Zeisel A, Turnbaugh PJ, Soen Y (2018). Combining 16S rRNA gene variable regions enables high-resolution microbial community profiling. Microbiome.

[53] Ley RE, Hamady M, Lozupone C, Turnbaugh PJ, Ramey RR, Bircher JS (2008). Evolution of mammals and their gut microbes. Science.

[54] Wexler HM (2007). Bacteroides: the good, the bad, and the nitty-gritty. Clin Microbiol Rev.

[55] Derrien M, Vaughan EE, Plugge CM, de Vos WM. Akkermansia muciniphila gen. nov., sp. nov., a human intestinal mucin-degrading bacterium. Int J Syst Evolut Microbiol 2004;54(5):1469–76. 10.1099/ijs.0.02873-0 15388697

[56] Belzer C, De Vos WM (2012). Microbes inside—from diversity to function: the case of Akkermansia. ISME J.

