# Supplementary material for: Classification and prediction of Mycobacterium Avium subsp. Paratuberculosis (MAP) shedding severity in cattle based on young stock heifer faecal microbiota composition using random forest algorithms
Source: Anim Microbiome. 2021 Nov 14;3:78. doi: 10.1186/s42523-021-00143-y (PMC8591832; doi:10.1186/s42523-021-00143-y)
Supplement: Supplementary file 4 — Additional file 4: Shedding intensity score calculation. [file 42523_2021_143_MOESM4_ESM.pdf]

Additional files 1

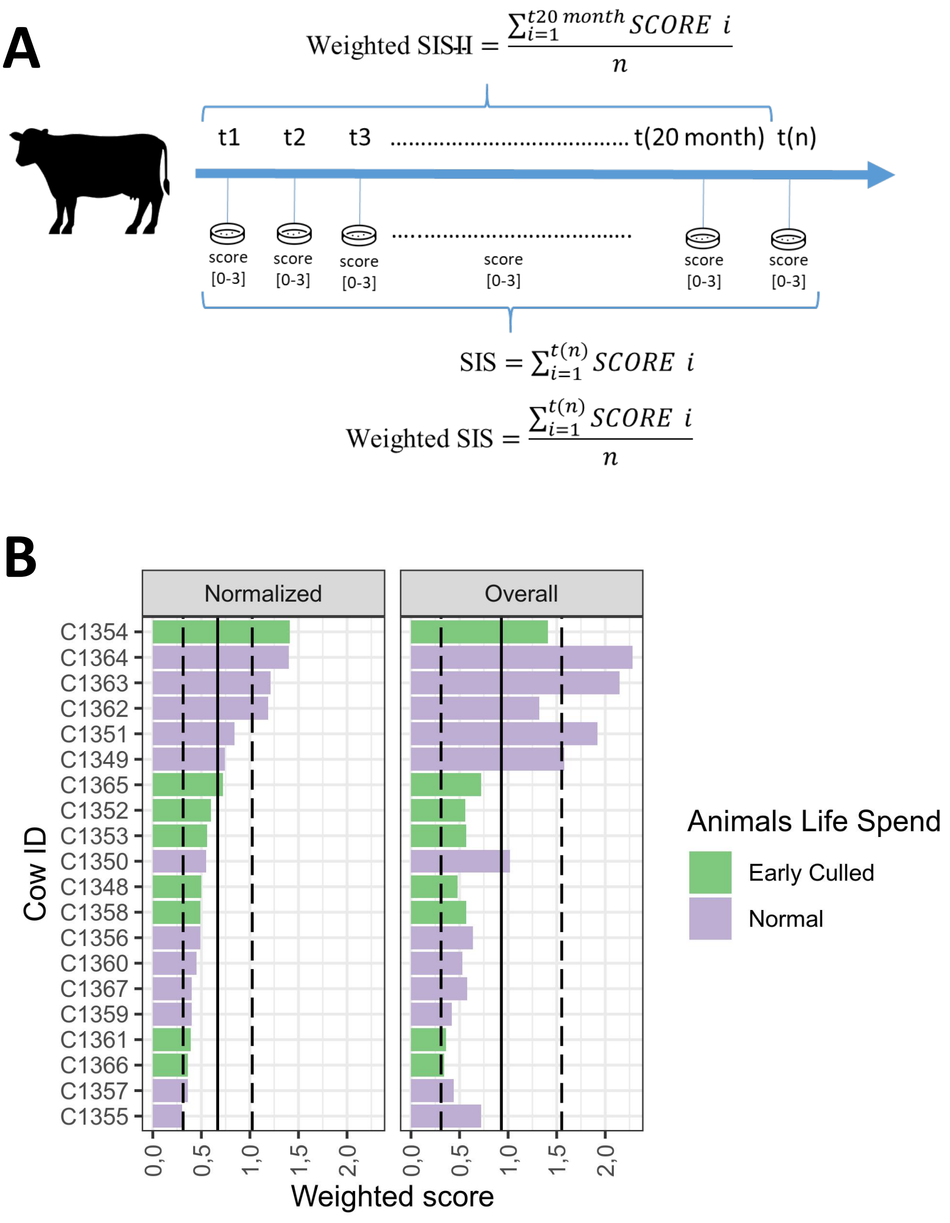

Figure S1. **A)** Schematic representation of the Shedding Intensity Score (SIS), Weighted SIS and Weighted SISII calculation process. **B)** Bar chart of Weighted Shedding Intensity Scores (SIS) calculated based on samples from the first 20 month of experiment (Normalized) and all available data (Overall). The solid blue line marked the average weighted SIS and dashed lines marked 0.5 of Standard deviation.

Table S1. Number of samples per cow used for microbiota profiling

Cow ID	Samples (n)	Cow ID	Samples (n)	Cow ID	Samples (n)	Cow ID	Samples (n)
**C1348**	11	**C1353**	9	**C1358**	8	**C1363**	19
**C1349**	10	**C1354**	9	**C1359**	20	**C1364**	18
**C1350**	12	**C1355**	12	**C1360**	22	**C1365**	8
**C1351**	15	**C1356**	19	**C1361**	9	**C1366**	9
**C1352**	9	**C1357**	12	**C1362**	13	**C1367**	13

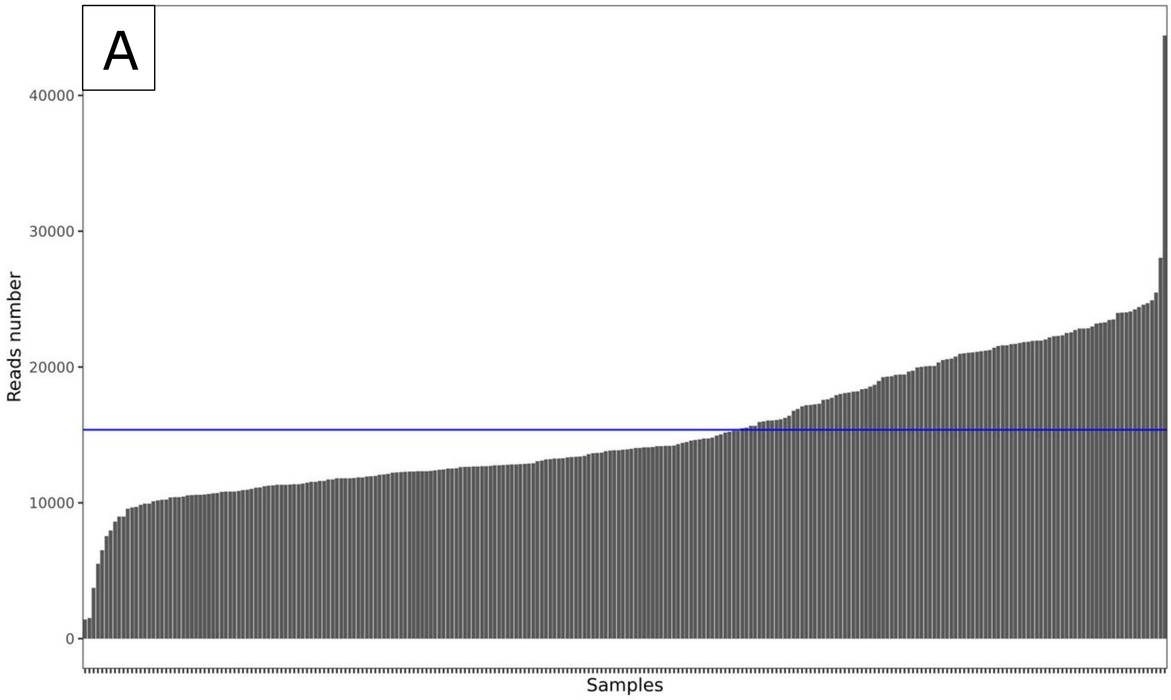

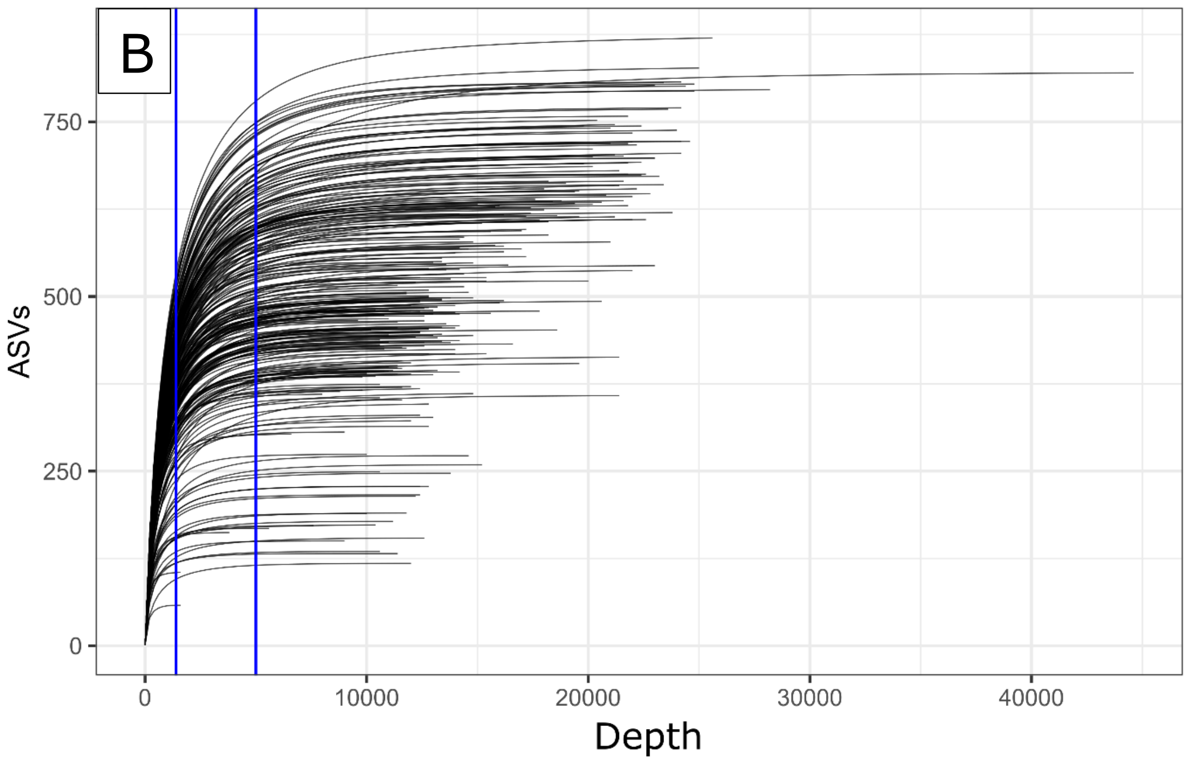

Figure S2. A) Number of filtered reads per sample. Blue line shows the median value. **B)** Rarefaction curves based on number observed species ASVs at a given sequencing depth. Black lines represent individual samples and vertical blue lines represent 1000 bp and 5000 bp sequencing depth.

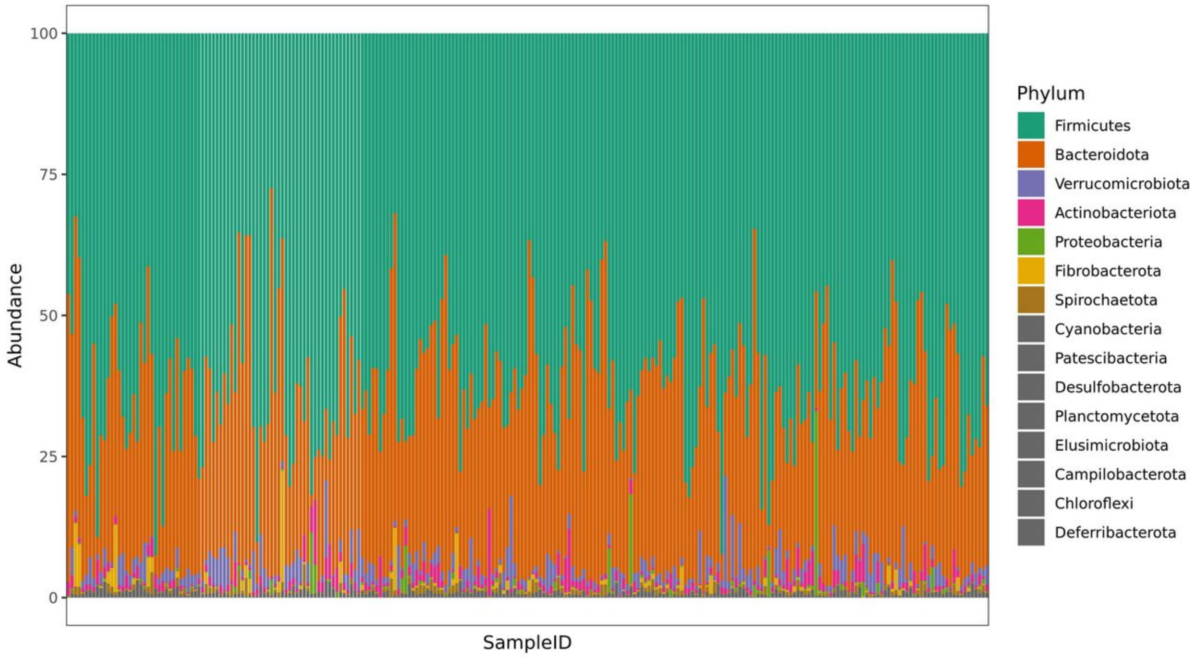

Figure S3. Barplot represents microbial phylogenetic composition on phylum level expressed in relative abundance.

Table S2. Summary information about relative abundance of microbial phyla detected across the samples. CI interval calculated at 95%.

Phylum	1st Qu.	3rd Qu.	Max.	Median	Min.	Estimate	CI lower	CI upper	Std. Error
Actinobacteriota	0.46	1.68	15.5	0.88	0	1.47	1.25	1.7	0.12
Bacteroidota	21.9	37.26	68.85	30.36	2.15	30.67	29.18	32.17	0.76
Campilobacterota	0	0	0.16	0	0	0.01	0	0.01	0
Chloroflexi	0	0	0.22	0	0	0	0	0.01	0
Cyanobacteria	0.13	0.45	1.42	0.27	0	0.31	0.28	0.35	0.02
Deferribacterota	0	0	0.03	0	0	0	0	0	0
Desulfobacterota	0	0.15	1.2	0.04	0	0.13	0.1	0.16	0.01
Elusimicrobiota	0	0	1.07	0	0	0.02	0.01	0.02	0
Fibrobacterota	0	0.3	19.74	0.07	0	0.63	0.38	0.87	0.12
Firmicutes	56.39	71.3	92.47	63.28	27.41	62.78	61.32	64.25	0.74
Patescibacteria	0.06	0.3	2.3	0.15	0	0.28	0.23	0.32	0.02
Planctomycetota	0.03	0.19	0.82	0.1	0	0.13	0.11	0.15	0.01
Proteobacteria	0.07	0.34	32.81	0.16	0	0.63	0.31	0.94	0.16
Spirochaetota	0.11	0.78	4.11	0.31	0	0.58	0.49	0.67	0.04
Verrucomicrobiota	0.63	3.11	19.94	1.56	0	2.36	2.03	2.7	0.17

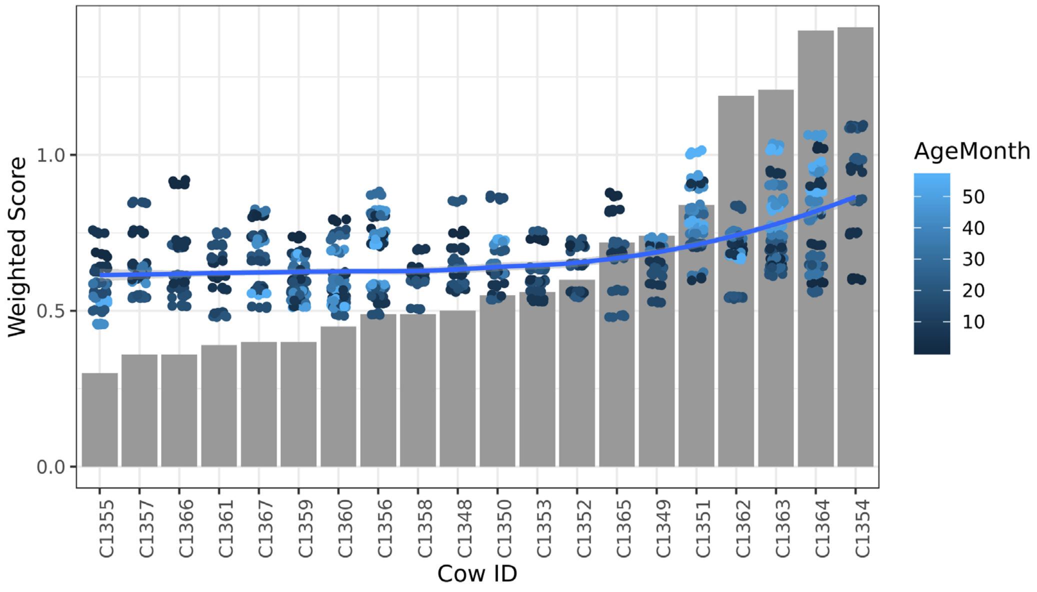

Figure S4. Prediction of Weighted Shedding Intensity Scores (SIS) using Random Forest (RF) regression model. Gray bars represent actual shedding score per animal and blue dots show Weighted SIS predicted by RF model. The blue line represents the regression line fitted using LOESS method.

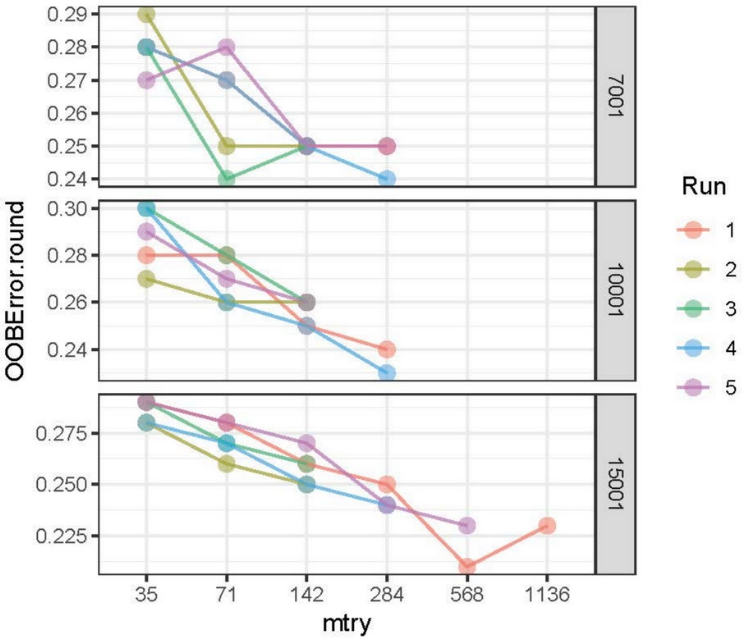

Figure S5. Parameters optimization of the General RF model. The graph shows the relationship between model errors and number of features used at a split (mtry parameter). Each facet shows the number of trees used in an optimization effort. Each combination of parameters (mtry and ntree) was tested five times, and each test run reflected by colour of dots and lines.

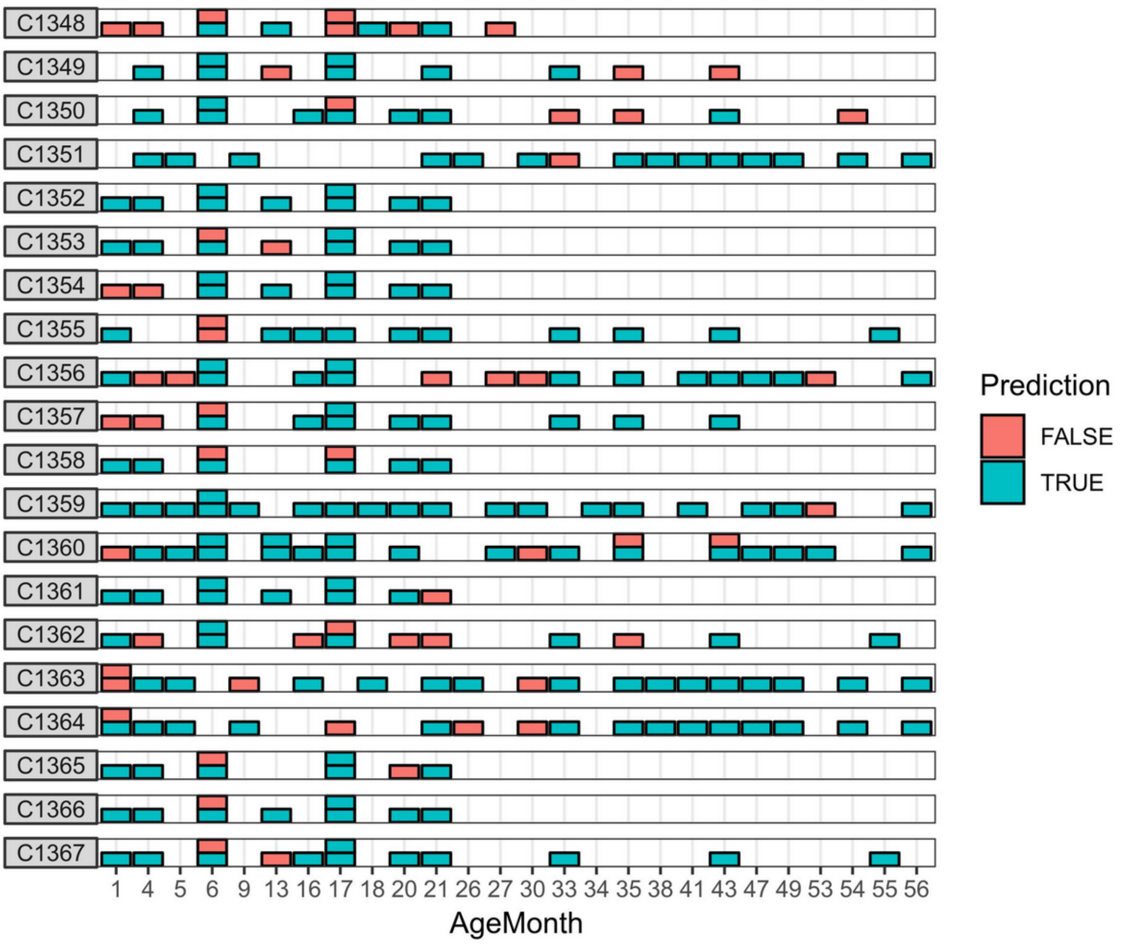

Figure S6. Results of samples classification when all samples from a single animal are used as the "validation" dataset and rest as the "training" dataset.
